# Attempted Suicide in Bipolar Disorder: Risk Factors in a Cohort of 6086 Patients

**DOI:** 10.1371/journal.pone.0094097

**Published:** 2014-04-04

**Authors:** Dag Tidemalm, Axel Haglund, Alina Karanti, Mikael Landén, Bo Runeson

**Affiliations:** 1 Department of Clinical Neuroscience, Center for Psychiatric Research, Karolinska Institutet, Stockholm, Sweden; 2 Institute of Neuroscience and Physiology, Sahlgrenska Academy at University of Gothenburg, Sweden; 3 Department of Medical Epidemiology and Biostatistics, Karolinska Institutet, Stockholm, Sweden; Chiba University Center for Forensic Mental Health, Japan

## Abstract

**Objective:**

Bipolar disorder is associated with high risk of self-harm and suicide. We wanted to investigate risk factors for attempted suicide in bipolar patients.

**Method:**

This was a cohort study of 6086 bipolar patients (60% women) registered in the Swedish National Quality Register for Bipolar Disorder 2004–2011 and followed-up annually 2005–2012. Logistic regression was used to calculate adjusted odds ratios for fatal or non-fatal attempted suicide during follow-up.

**Results:**

Recent affective episodes predicted attempted suicide during follow-up (men: odds ratio = 3.63, 95% CI = 1.76–7.51; women: odds ratio = 2.81, 95% CI = 1.78–4.44), as did previous suicide attempts (men: odds ratio = 3.93, 95% CI = 2.48–6.24; women: odds ratio = 4.24, 95% CI = 3.06–5.88) and recent psychiatric inpatient care (men: odds ratio = 3.57, 95% CI = 1.59–8,01; women: odds ratio = 2.68, 95% CI = 1.60–4.50). Further, those with many lifetime depressive episodes were more likely to attempt suicide. Comorbid substance use disorder was a predictor in men; many lifetime mixed episodes, early onset of mental disorder, personality disorder, and social problems related to the primary group were predictors in women.

**Conclusion:**

The principal clinical implication of the present study is to pay attention to the risk of suicidal behaviour in bipolar patients with depressive features and more severe or unstable forms of the disorder.

## Introduction

Several risk factors for suicidal behaviour have been identified, including mental disorder, history of suicidal behaviour, aggression or impulsivity, family history of suicidal behaviour, social isolation, marital problems, work problems, and poor physical health [1]–[5]. Among common mental disorders, bipolar affective disorder implies a particular risk of both non-fatal self-harm and completed suicide [6], [7]. Bipolar disorder is characterized by manic and depressive (including mixed) episodes of varying severity; it is a serious and often disabling mood disorder associated with reduced life expectancy [8], [9]. A review has estimated the risk of suicide in bipolar patients to be 20–30 times higher than that of the general population [10]. The risk is greater among those who have been admitted to inpatient care due to bipolar disorder [11] and especially high in bipolar patients admitted to inpatient care after attempted suicide [12]. Recent studies from Denmark [13] and the UK [14] included bipolar outpatients as well as inpatients. These studies show that the risk of suicide in bipolar disorder, though high, has probably been often overestimated due to a focus on more severely ill, hospital-treated, patients. The Danish study found 5% suicides among the women and 8% among the men in those with bipolar disorder in a long-term follow-up of patients after psychiatric contact [13]. The lifetime prevalence of attempted suicide in bipolar patients has been estimated to 34% in women and 19% in men [15].

Generally, the patterns of suicidal behaviour vary between the genders, with higher rates of attempted suicide in women and higher rates of completed suicide in men [3]. These gender differences in suicidal behaviour may, however, be less pronounced in subjects with bipolar disorder [6]. Reviews have found specific risk factors for suicidal behaviour in bipolar disorder, for instance early onset of the disorder [6], [16]. Also, it has been suggested that the risk of suicide is higher in a bipolar type 2 than in type 1 disorder [17], [18]. To date it has proven difficult to assess the relative weight of individual risk factors [19].

More detailed knowledge of risk factors in this high-risk group may inform suicide preventive measures. Clinical studies often investigate samples of limited size and are thus underpowered for the purpose. On the other hand, previous studies based on large national patient registers have sufficient statistical power but lack comprehensive clinical data. The Swedish national quality register for bipolar disorder contains detailed clinical data for a large number of patients and may thus provide information necessary for comparing clinical risk factors [20].

We wanted to study risk factors for attempted suicide in bipolar disorder with clinical data from a patient sample large enough for comparisons of weight or severity of risk factors. Our hypotheses were that recent affective episodes, recent psychiatric inpatient care, early onset of psychiatric problems, family history of affective disorder, comorbidity, complicating social factors, and violent behaviour predict suicide attempts in bipolar patients, and also that risks of attempted suicide differ between subtypes of bipolar disorder.

## Material and Methods

### Ethics statement

Ethical approval was obtained from the Gothenburg Regional Ethics Committee (294-11).

### Data source and sample

This was a national cohort study using clinical data from the Swedish National Quality Register for Bipolar Disorder (BipoläR). BipoläR is one of several somatic and psychiatric national quality assurance registers established in the Swedish health care system in recent decades [21], [22]. It contains detailed individual data for patients diagnosed with bipolar disorder type 1 (ICD-10 codes F30.1-30.9, F31.0-31.7), type 2 (F31.8), NOS (not otherwise specified) (F31.9), or schizoaffective disorder of bipolar type (F25.0). The register comprises a large number of variables with data on psychiatric and somatic morbidity (including comorbidity) as well as anamnestic and social factors. The register includes both patients treated exclusively in outpatient care and others with more severe forms of the disorder requiring hospital treatment. Data on each patient are recorded in the register at a first registration; additional data on the individuals are then collected at continuous annual follow-ups. Participation in the register is voluntary for patients and clinicians. The data are collected by psychiatrists and staff specifically trained in the diagnosis and treatment of bipolar disorder and with access to all clinical data of each patient. Consequently, both the data quality in general and the validity of bipolar diagnoses in the BipoläR are likely to be high. More details about the register have been described elsewhere [20].

The study population was 6086 bipolar patients registered in the BipoläR 2004–2011 and followed-up annually 2005–2012; the mean total follow-up time was 2.4 years (SD = 1.3). The cohort consisted of 2408 men and 3678 women, mean age 49.3 years (SD = 12.8) and 48.3 years (SD = 13.0), respectively. Data were extracted in December 2012.

### Variables and statistical analyses

The outcome variable was attempted suicide during follow-up, as registered in the BipoläR and defined as one or more fatal or non-fatal suicide attempts during the year before each annual follow-up date. The definition of attempted suicide in the BipoläR corresponds to the ICD-10 category *Intentional self-harm* (codes X60-84). Bivariate analyses of the following explanatory variables registered at baseline were conducted in two-by-two tables with the chi-square test and Fisher's exact test: previous suicide attempts (lifetime), bipolar disorder subtype (type 1, type 2, NOS, and schizoaffective disorder of affective type), affective episodes during the year before baseline, many (4+) specified lifetime affective episodes (depressive, hypomanic, manic, or mixed), family history of affective disorder (bipolar disorder, unipolar disorder, or dysthymia in first-degree relatives), psychiatric inpatient care during the year before baseline (any mental disorder), early onset of psychiatric problems (before 18 years of age; any mental disorder), psychiatric comorbidity, complicating somatic disorder (e.g., illness, injury, or poisoning), complicating social factors (i.e., family-, work-, or economy-related), and violent behaviour (directed towards other people). The explanatory variables that predicted attempted suicide in the bivariate analyses were included in a multiple logistic regression model, together with the potential confounder age. Adjusted odds ratios with 95% confidence interval (95% CI) were computed. A second multiple logistic regression model was used to analyse subcategories of complicating social factors. This model was similar to the first one, but included social factors related to the primary group, social environment, school, work, housing, economy, healthcare, or criminal behaviour. Previous suicide attempts may be on the causal pathway from the other studied risk factors; therefore, this variable was only treated as an explanatory variable and not as a covariate to be adjusted for. Further, the generally important variables of functional level and educational level could both be on the causal pathway from bipolar disorder to suicidal behaviour. Therefore, these variables were not regarded as potential confounders and were not included in multiple regression models. In BipoläR, the variables of comorbid somatic disorder and social factors are specifically defined as factors with a potentially complicating effect on psychiatric treatment. The comorbid psychiatric conditions studied were substance use disorder (ICD-10 codes F10-19; F55), nonorganic psychoses (F20-29 except F25.0; F53.1), anxiety disorders (F40-48; F62), eating disorders (F50), and personality disorders (F60-F61; F68.8). The results were stratified by gender, except when gender was used as an explanatory variable. Data for the following variables were only available for subsets of the cohort: recent psychiatric inpatient care (n = 2094), early onset of psychiatric problems (n = 3704), family history of affective disorder (n = 3348), and violent behaviour (n = 3912). SPSS (version 22.0) was used for all statistical analyses.

## Results

Table 1 (men) and Table 2 (women) show descriptive and bivariate statistics as well as odds ratios from the multiple logistic regressions for attempted suicide during follow-up. Thirteen fatal and 338 non-fatal suicide attempts occurred during follow-up (data not shown in table). The proportion of attempted suicide during follow-up was significantly higher in women than in men (6.9% compared with 4.1%; χ2 = 20.61, df = 1, p<0.001; χ2 result not shown in table). The variables of many lifetime manic episodes, family history of affective disorder, and presence of complicating somatic disorder at baseline did not increase the risk of attempted suicide in bivariate analysis. These variables were thus not included in multiple regression models. All other explanatory variables showed significant differences for men, women, or both genders in the bivariate analyses.

**Table 1:** Statistics for attempted suicide during follow-up among 2408 male bipolar patients.

		N	Suicide attempts^a^		?2 b	p-value	Odds ratio^c^	95% CI
			N	%				
Previous suicide attempts at baseline (lifetime)	Yes	689	66	9.6	73.07	<0.001	3.93	2.48–6.24
	No	1719	32	1.9				
Bipolar disorder subtype^d^	Type 1	1242	45	3.6	Referent			
	Type 2	795	35	4.4	0.59	0.443	0.96	0.59–1.55
	NOS^e^	291	18	6.2	3.31	0.069	1.65	0.91–3.00
	SADB^f^	80	0	0		0.107^g^		
Affective episodes during the year before baseline	Yes	1395	89	6.4	43.93	<0.001	3.63	1.76–7.51
	No	1013	9	0.9				
≥4 lifetime depressive episodes	Yes	1612	84	5.2	15.39	<0.001	2.06	1.08–3.92
	No	796	14	1.8				
≥4 lifetime hypomanic episodes	Yes	1148	65	5.7	13.48	<0.001	1.30	0.80–2.12
	No	1260	33	2.6				
≥4 lifetime manic episodes	Yes	602	30	5.0	1.42	0.234		
	No	1806	68	3.8				
≥4 lifetime mixed episodes	Yes	400	29	7.3	11.47	<0.001	1.18	0.72–1.96
	No	2008	69	3.4				
Family history of affective disorder^h^	Yes	707	40	5.7	2.50	0.114		
	No	627	23	3.7				
Psychiatric inpatient care during the year before baseline^i^	Yes	142	15	10.6		<0.001^g^	3.57	1.59–8.01
	No	695	13	1.9				
Early onset of psychiatric problems^j^	Yes	510	38	7.5	13.89	<0.001	1.55	0.88–2.74
	No	931	28	3.0				
Psychiatric comorbidity								
Substance use disorder	Yes	189	20	10.6	20.51	<0.001	1.95	1.11–3.44
	No	2219	78	3.5				
Nonorganic psychosis	Yes	12	1	8.3		0.393^g^		
	No	2396	97	4.0				
Anxiety disorder	Yes	196	20	10.2	18.89	<0.001	1.50	0.85–2.66
	No	2212	78	3.5				
Eating disorder	Yes	11	3	27.3		<0.01^g^	5.09	1.07–24.33
	No	2397	95	4.0				
Personality disorder	Yes	50	4	8.0		0.144^g^		
	No	2358	94	4.0				
Complicating somatic disorder^k^	Yes	676	25	3.7	0.21	0.644		
	No	1732	73	4.2				
Complicating social factors^l^	Yes	639	50	7.8	30.12	<0.001	1.67	1.08–2.59
	No	1769	48	2.7				
Violent behaviour^m^	Yes	305	24	7.9	10.11	<0.01	1.42	0.81–2.47
	No	1227	43	3.5				
Total		2408	98	4.1				

Distribution of study variables, bivariate statistics, and adjusted odds ratios with 95% confidence interval (95% CI) for attempted suicide during follow-up among 2408 male bipolar patients recorded in the BipoläR 2004–2011 and followed-up annually 2005–2012.

a Fatal or non-fatal attempted suicide during follow-up, as registered in the BipoläR at annual follow-ups.

b Chi-square values derived from two-by-two tables, hence df = 1.

c When previous suicide attempts was explanatory variable, the logistic regression model included the potential confounder age as well as all variables in the table that had a significant p-value in bivariate comparison. When other variables in the table were explanatory variables, the variable previous suicide attempts was omitted.

d Categorical variable. Bipolar disorder type 1 was reference category in the chi-square tests and in the multiple regression.

e Bipolar disorder of unspecified type.

f Schizoaffective disorder of bipolar type.

g One cell had expected count less than 5; therefore, Fisher's exact test was used instead of the chi-square test.

h First-degree relatives; data available for a subset of the cohort only.

i Data available for a subset of the cohort only.

j Before 18 years of age; data available for a subset of the cohort only.

k For instance, illness, injury, or poisoning.

l For instance, family-, work- or economy-related.

m Directed towards people; data available for a subset of the cohort only.

**Table 2:** Statistics for attempted suicide during follow-up among 3678 female bipolar patients.

		N	Suicide attempts^a^		?2 b	p-value	Odds ratio^c^	95% CI
			N	%				
Previous suicide attempts at baseline (lifetime)	Yes	1451	199	13.7	173.07	<0.001	4.24	3.06–5.88
	No	2227	54	2.4				
Bipolar disorder subtype^d^	Type 1	1639	89	5.4	Referent			
	Type 2	1410	116	8.2	9.01	<0.01	1.07	0.78–1.46
	NOS^e^	524	43	8.2	4.87	<0.05	1.08	0.71–1.62
	SADB^f^	105	5	4.8	0.01	0.943	0.90	0.34–2.38
Affective episodes during the year before baseline	Yes	2359	229	9.7	80.95	<0.001	2.81	1.78–4.44
	No	1319	24	1.8				
≥4 lifetime depressive episodes	Yes	2764	222	8.0	22.37	<0.001	1.93	1.27–2.94
	No	914	31	3.4				
≥4 lifetime hypomanic episodes	Yes	1741	145	8.3	10.42	<0.01	0.91	0.67–1.24
	No	1937	108	5.6				
≥4 lifetime manic episodes	Yes	810	53	6.5	0.12	0.727		
	No	2868	200	7.0				
≥4 lifetime mixed episodes	Yes	728	85	11.7	31.68	<0.001	1.40	1.02–1.90
	No	2950	168	5.7				
Family history of affective disorder^g^	Yes	1151	90	7.8	0.90	0.342		
	No	863	57	6.6				
Psychiatric inpatient care during the year before baseline^h^	Yes	222	32	14.4	35.75	<0.001	2.68	1.60–4.50
	No	1035	40	3.9				
Early onset of psychiatric problems^i^	Yes	919	122	13.3	69.60	<0.001	1.50	1.01–2.23
	No	1344	50	3.7				
Psychiatric comorbidity								
Substance use disorder	Yes	146	15	10.3	2.21	0.137		
	No	3532	238	6.7				
Nonorganic psychosis	Yes	47	7	14.9		<0.05^j^	1.50	0.60–3.79
	No	3631	246	6.8				
Anxiety disorder	Yes	433	53	12.2	21.09	<0.001	1.25	0.88–1.78
	No	3245	200	6.2				
Eating disorder	Yes	106	25	23.6	44.91	<0.001	1.85	1.11–3.09
	No	3572	228	6.4				
Personality disorder	Yes	122	29	23.8	53.51	<0.001	2.29	1.42–3.69
	No	3556	224	6.3				
Complicating somatic disorder^k^	Yes	1252	84	6.7	0.05	0.824		
	No	2426	169	7.0				
Complicating social factors^l^	Yes	1115	127	11.4	49.83	<0.001	1.55	1.17–2.04
	No	2563	126	4.9				
Violent behaviour^m^	Yes	248	29	11.7	6.41	<0.05	0.93	0.59–1.48
	No	2129	149	7.0				
Total		3678	253	6.9				

Distribution of study variables, bivariate statistics, and adjusted odds ratios with 95% confidence interval (95% CI) for attempted suicide during follow-up among 3678 female bipolar patients recorded in the BipoläR 2004–2011 and followed-up annually 2005–2012.

a Fatal or non-fatal attempted suicide during follow-up, as registered in the BipoläR at annual follow-ups.

b Chi-square values derived from two-by-two tables, hence df = 1.

c When previous suicide attempts was explanatory variable, the logistic regression model included the potential confounder age as well as all variables in the table that had a significant p-value in bivariate comparison. When other variables in the table were explanatory variables, the variable previous suicide attempts was omitted.

d Categorical variable. Bipolar disorder type 1 was reference category in the chi-square tests and in the multiple regression.

e Bipolar disorder of unspecified type.

f Schizoaffective disorder of bipolar type.

g First-degree relatives; data available for a subset of the cohort only.

h Data available for a subset of the cohort only.

i Before 18 years of age; data available for a subset of the cohort only.

j One cell had expected count less than 5; therefore, Fisher's exact test was used instead of the chi-square test.

k For instance, illness, injury, or poisoning.

l For instance, family-, work- or economy-related.

m Directed towards people; data available for a subset of the cohort only.

**Figure 1:**
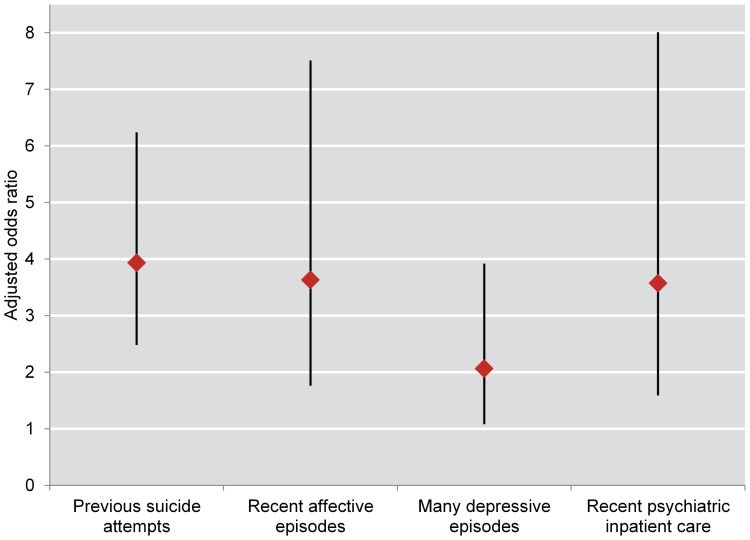
Odds ratios for attempted suicide during follow-up among 2408 male bipolar patients. Adjusted odds ratios with 95% confidence interval for fatal or non-fatal attempted suicide during follow-up among 2408 male bipolar patients recorded in the BipoläR 2004–2011 and followed-up annually 2005–2012.

**Figure 2:**
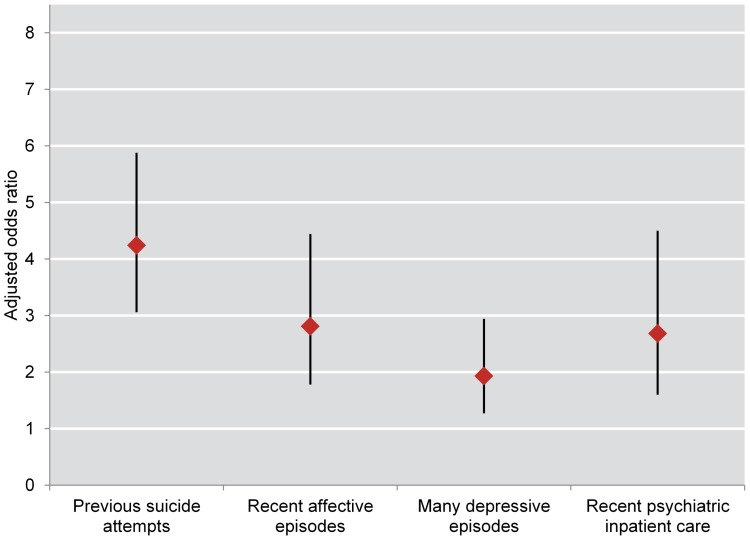
Odds ratios for attempted suicide during follow-up among 3678 female bipolar patients. Adjusted odds ratios with 95% confidence interval for fatal or non-fatal attempted suicide during follow-up among 3678 female bipolar patients recorded in the BipoläR 2004–2011 and followed-up annually 2005–2012.

In the multiple regression analysis, women had higher adjusted odds for attempted suicide than men during follow-up (odds ratio = 1.40, 95% CI = 1.09–1.81) (data not shown in table). Previous suicide attempts at baseline (men: odds ratio = 3.93, 95% CI = 2.48–6.24; women: odds ratio = 4.24, 95% CI = 3.06–5.88), affective episodes during the year before baseline (men: odds ratio = 3.63, 95% CI = 1.76–7.51; women: odds ratio = 2.81, 95% CI = 1.78–4.44), many lifetime depressive episodes (men: odds ratio = 2.06, 95% CI = 1.08–3.92; women: odds ratio = 1.93, 95% CI = 1.27–2.94), and psychiatric inpatient care during the year before baseline (men: odds ratio = 3.57, 95% CI = 1.59–8.01; women: odds ratio = 2.68, 95% CI = 1.60–4.50) all predicted suicide attempt during follow-up (Table 1 and Table 2; Figure 1 and Figure 2).

The odds were significantly increased only among women for many lifetime mixed episodes (odds ratio = 1.40, 95% CI = 1.02–1.90) and early onset of psychiatric problems (odds ratio = 1.50, 95% CI = 1.01–2.23). The complicating social factors variable was a predictor in the multiple analysis (men: odds ratio = 1.67, 95% CI = 1.08–2.59; women: odds ratio = 1.55, 95% CI = 1.17–2.04), as was comorbid eating disorder (men: odds ratio = 5.09, 95% CI = 1.07–24.33; women: odds ratio = 1.85, 95% CI = 1.11–3.09). Comorbid substance use disorder was a predictor in men (odds ratio = 1.95, 95% CI = 1.11–3.44), while comorbid personality disorder predicted suicide attempt in women (odds ratio = 2.29, 95% CI = 1.42–3.69). Analyses of subcategories of complicating social factors showed further differences between men and women. The odds for economy-related social problems were significantly increased in both genders (men: odds ratio = 2.09, 95% CI = 1.18–3.69; women: odds ratio = 1.58, 95% CI = 1.08–2.32), but social problems related to the primary group predicted attempted suicide in women only (odds ratio = 1.60, 95% CI = 1.15–2.24), (data not shown in table).

Non-significantly increased odds ratios were found for a number of variables, for instance, anxiety disorder (both genders), nonorganic psychosis (women), early onset of psychiatric problems (men), and violent behaviour (men). Bipolar disorder type 1 and schizoaffective disorder of bipolar type had non-significantly lower proportions of attempted suicide during follow-up than the other subtypes.

## Discussion

The proportion of attempted suicide in the cohort was high during follow-up; the strongest predictors in the multiple regression were affective episodes during the year before baseline, previous suicide attempts, and psychiatric inpatient care during the year before baseline. Those who had had many lifetime depressive episodes were more likely to attempt suicide than those who had not; there was no such difference between those with and without many lifetime manic episodes. The multiple regression analysis also identified other predictors, which were of less magnitude and differed between the genders. Many lifetime mixed episodes, early onset of psychiatric problems, and personality disorder were predictors in women, whereas comorbid substance use disorder was a predictor in men. Further, social problems related to the primary group were a predictor of attempted suicide in women. Contrary to our hypotheses, family history of affective disorder, complicating somatic factors, and violent behaviour did not predict suicide attempt during follow-up.

Before discussing the results further, some strengths and limitations of the study should be mentioned. The present study was based on highly valid patient data from a Swedish clinical register for bipolar disorder. Bipolar patients with varying degrees of severity of illness were included. This serves to avoid the bias inherent in samples of inpatients, where a larger proportion are severely ill and probably a substantial proportion were admitted due to high suicide risk. Also, the large sample size was adequate for adjusted comparisons relevant to our hypotheses, for stratifying the results by gender and, importantly, for including several risk factors in the same model. We assume, therefore, that the calculated estimates in the study are reliable. Nevertheless, an even larger sample size would be needed to discern whether some of our non-significant results were due to insufficient power. Also, since the follow-up time was relatively short (mean = 2.4 years), it was not feasible to study completed suicide, due to the rarity of that phenomenon. Participation in the BipoläR is voluntary for the patients and it is so far unknown whether some specific categories of bipolar patients are more likely to participate. Therefore, the possibility of some inclusion bias cannot be ruled out. Further, some potentially important information was lacking in our data, for instance, on the severity of symptoms at baseline, increasing severity of episodes, or family history of suicidal behaviour, the latter two of which are known important factors [6], [23], [24]. Finally, we did not have data on details of each suicide attempt, for instance the exact date or the method used.

As many others have found, a previous suicide attempt is a strong (possibly the strongest) risk factor for suicidal behaviour [2]–[4], [25], not least in bipolar patients [6], [12], [17], [18], [26]. In this study, however, recent affective episodes during the year before baseline was an almost equally strong predictor. Also, supporting the findings from other studies [6], [17], [26]–[28], the odds for attempted suicide were higher in those with many depressed or mixed episodes but not in those with many manic episodes. These results point to the fundamental importance of observing signs of depressive symptomatology and supplying adequate treatment, in the follow-up of bipolar patients. Evidence-based treatment together with stable access to care in specific programs for bipolar patients has been shown to yield low rates of completed suicide [29].

Relational and economic stressors have previously been identified as risk factors for suicidal behaviour in those with bipolar disorder [17], [18], as have comorbid substance abuse [6], [17], eating disorder [6] and anxiety disorder [6]. Personality disorder is generally a known risk factor for suicidal behaviour [2]–[4]. In the present study of bipolar patients, however, some of the social factors and comorbid psychiatric conditions predicted suicide attempt only in women or only in men. This distinct difference between the risk patterns of men and women is, to our knowledge, a novel finding. Comorbid substance use disorder doubled the risk of subsequent suicidal behaviour in men with bipolar affective disorder. Substance use disorder as a primary disorder is in itself a risk factor for suicide [2], [30], also in subjects who have attempted suicide [12]. Substance use as a comorbid disorder is certainly a sign of risk, as comorbidity *per se* is a risk factor for suicide [2]. The diagnosis of substance use should thus warrant attention in the treatment of male patients with bipolar disorder.

In accordance with two reviews [6], [31] the differences in risk of suicidal behaviour between subtypes of bipolar disorder were not significant in the present study. In contrast to these findings, however, other reviews found a higher risk of suicidal behaviour for bipolar disorder type 2 [16]–[18]. It should be noted that attempted suicide was the only outcome variable in our study; the difference between bipolar disorder type 1 and type 2 may prove larger for completed suicide in future follow-ups of the same cohort. The specific features of recent episodes may be more important than bipolar subtype for the risk of suicidal behaviour. For instance, the intensity of depressive episodes, the severity of episodes [6], or depression with atypical features [32] could be more relevant. The results of the present study point to the importance of severity of disorder, indicated by early onset (significant in women only) and need of psychiatric inpatient care.

In line with results from one review [17] but in contrast to another [6], women had significantly higher odds for attempted suicide in the adjusted analyses. Taken together, results from our study and the two cited reviews seem inconclusive as to whether bipolar disorder increases the risk of attempted and completed suicide to a substantially different degree in men and women.

## Conclusions

Results from this large cohort study of bipolar patients show that previous suicidal behaviour and recent affective episodes may be the strongest predictors of suicidal behaviour in bipolar patients. Several previous depressive and mixed episodes could also be important predictors. Other factors should also be taken into account in suicide risk assessment and prevention, like comorbid substance use in men, or general severity of disorder. It is possible that risk factor patterns differ between men and women; more studies comparing male and female bipolar patients in this regard are needed. The principal clinical implication of the present study should be to pay attention to the risk of suicidal behaviour in bipolar patients with depressive features and more severe or unstable forms of the disorder.
